# Possible Immune Regulation of Natural Killer T Cells in a Murine Model of Metal Ion-Induced Allergic Contact Dermatitis

**DOI:** 10.3390/ijms17010087

**Published:** 2016-01-12

**Authors:** Kenichi Kumagai, Tatsuya Horikawa, Hiroaki Shigematsu, Ryota Matsubara, Kazutaka Kitaura, Takanori Eguchi, Hiroshi Kobayashi, Yasunari Nakasone, Koichiro Sato, Hiroyuki Yamada, Satsuki Suzuki, Yoshiki Hamada, Ryuji Suzuki

**Affiliations:** 1 Department of Oral and Maxillofacial Surgery, School of Dental Medicine, Tsurumi University, 2-3-1 Tsurumi, Tsurumi-ku, Yokohama 230-8501, Japan; kumagai-kenichi@tsurumi-u.ac.jp (K.Ku.); shigematsu-h@tsurumi-u.ac.jp (H.S.); matsubara-ryota@tsurumi-u.ac.jp (R.M.); from7ritoyou@gmail.com (Y.N.); sato-ki@tsurumi-u.ac.jp (K.S.); hamada-y@tsurumi-u.ac.jp (Y.H.); 2 Department of Rheumatology and Clinical Immunology, Clinical Research Center for Rheumatology and Allergy, Sagamihara National Hospital, National Hospital Organization, 18-1 Sakuradai, Minami-ku, Sagamihara 252-0392, Japan; k-kitaura@sagamihara-hosp.gr.jp (K.Ki.); fhb19830419@yahoo.co.jp (T.E.); hirok0614@yahoo.co.jp (H.K.); 3 Department of Dermatology, Nishi-Kobe Medical Center, 5-7-1 Kojidai, Kobe 651-2273, Japan; thorikaw@med.kobe-u.ac.jp; 4 Department of Oral and Maxillofacial Surgery, Toshiba Rinkan Hospital, 7-9-1 Kamitsuruma, Minami-ku, Sagamihara 252-0385, Japan; 5 Department of Oral and Maxillofacial Surgery, Shonan Tobu Hospital, 500 Nishikubo, Chigasaki 253-0083, Japan; 6 Division of Oral Maxillofacial Surgery, Department of Reconstructive Oral and Maxillofacial Surgery, Iwate Medical University School of Dentistry, Morioka, Iwate 020-8505, Japan; yamadah@iwate-med.ac.jp; 7 Section of Biological Sciences, Research Center for Odontology, The Nippon Dental University School of Life Dentistry at Tokyo, 1-9-20 Fujimi, Chiyoda-ku, Tokyo 102-8159, Japan; satsukis@tky.ndu.ac.jp

**Keywords:** metal allergy, delayed-type hypersensitivity, natural killer T cells, metal ion, T cell receptor

## Abstract

Metal often causes delayed-type hypersensitivity reactions, which are possibly mediated by accumulating T cells in the inflamed skin, called irritant or allergic contact dermatitis. However, accumulating T cells during development of a metal allergy are poorly characterized because a suitable animal model is unavailable. We have previously established novel murine models of metal allergy and found accumulation of both metal-specific T cells and natural killer (NK) T cells in the inflamed skin. In our novel models of metal allergy, skin hypersensitivity responses were induced through repeated sensitizations by administration of metal chloride and lipopolysaccharide into the mouse groin followed by metal chloride challenge in the footpad. These models enabled us to investigate the precise mechanisms of the immune responses of metal allergy in the inflamed skin. In this review, we summarize the immune responses in several murine models of metal allergy and describe which antigen-specific responses occur in the inflamed skin during allergic contact dermatitis in terms of the T cell receptor. In addition, we consider the immune regulation of accumulated NK T cells in metal ion–induced allergic contact dermatitis.

## 1. Introduction

Metal allergy is categorized as a delayed-type hypersensitivity (DTH) reaction, and the number of patients with metal allergy has increased because metal is increasingly used in jewelry, surgical instruments, and dental restorations [1]. Traditionally, nickel (Ni), cobalt (Co), palladium (Pd), and chromium (Cr) have been reported as causal metals of allergic contact dermatitis (ACD).

Metal ions are thought to form highly defined geometricall but reversible coordination complexes with partner molecules within the body, thereby becoming antigens. Metal allergy is characterized by the recruitment of lymphocytes and inflammatory cells, including T cells, dendritic cells, granulocytes, and macrophages, to the site of allergic inflammation. The immune response of infiltrating T cells during the elicitation phase of DTH is thought to be autoreactive reaction [2], yet their antigen specificity for each metal has not been determined.

In this review, we summarize the immune responses in several murine models of metal allergy and which antigen-specific responses occur in the inflamed skin during ACD in terms of the T cell receptor (TCR). In addition, we consider the immune regulation of accumulated natural killer (NK) T cells in metal ion-induced ACD.

## 2. Establishment of Murine Models for Metal Allergy

Most studies of metal allergies have been performed *in vitro* using sensitized human lymphocytes, and it is believed that T cells are essential for metal allergy, as well as contact hypersensitivity to classical haptens [2]. However, the mechanism through which pathogenic T cells at the sites of allergic inflammation contribute to the development of metal allergy has not been explored. To examine the involvement of these accumulated T cells in metal allergy, the establishment of suitable animal models is desired.

Previous studies have generated several murine models of metal allergy in the inflamed ear [3,4]. They examined the effect of lipopolysaccharide (LPS) on allergies to Ni and other metals in mice, and concluded that LPS is a major inducer of metal allergies and potently promotes such allergies via innate immunity and histidine decarboxylase (HDC) induction in cells. LPS, a component of the cell walls of many Gram-negative bacteria, signals through a complex consisting of CD14, toll-like receptor (TLR) 4, and MD-2 [5], leading to secretion of pro-inflammatory cytokines and potent activation of the innate immune system [6]. LPS stimulates innate immunity via TLR4 and stimulates dendritic cells expressing TLRs, inducing maturation and modifying adaptive immunity. Recently, Schmidt *et al.* reported that Ni directly stimulates human TLR4, but not mouse TLR4, which may be crucial for the development of contact allergy [7].

In addition to ACD, skin exposure to metal without primary sensitization produces irritant contact dermatitis (ICD). ICD is a non-specific inflammatory dermatitis mainly mediated by the direct toxicity of chemicals into the skin, which triggers the activation of the innate immune system. ACD corresponds to a delayed-type hypersensitivity response, and skin inflammation is caused by antigen-specific T cells. However, the precise immune response both of the ICD and ACD has not been elucidated because of their coexistence with the DTH reaction. Previous murine models of metal allergy have not been used to investigate the differences between ICD and ACD, and have not revealed the histopathological features of the DTH response, such as spongiosis and liquefaction degeneration of the epidermis caused by intercellular edema and lymphocytic infiltration in the epidermis. Therefore, previous metal allergic murine models have not provided complete evidence of the metal ion-specific immune response.

Based on these previous reports, our novel murine models of allergies against metals such as Pd, Ni, and Cr were induced through sensitization by administration of metal chlorides and LPS into the mouse groin followed by challenge with several kinds of metal chlorides in the footpad of mice [8,9,10]. In our metal allergic mouse models, we found significant differences between ICD and ACD. The footpad swelling was measured every day after the challenge with metal chloride injection into the footpad. The footpad swelling reached maximum at one day after challenge in all mice. The footpad swelling at one day after challenge in ICD and ACD mice was similar. The footpad swelling was reduced at seven days after challenge in ICD mice. However, the footpad swelling continued for 14 days after challenge in ACD mice [10]. We found that both types of contact dermatitis could not be differentiated by the macroscopic appearance because footpad swelling was the same in both mouse models at one day after challenge. However, at seven days after challenge, inflammatory cells accumulated around the superficial venular bed and extended into the epidermis in ACD mice. Furthermore, the epidermal keratinocytes were partially separated, creating spongiotic dermatitis.

Regarding the skin pathophysiology in ACD, the inflammatory process is caused by a type-IV cell-mediated reaction occurring in a sensitized individual after renewed contact with the antigen. In the acute stage of ACD, the spongiosis and vesicle formation are mainly observed histologically. In subacute eczema, acanthosis increases with the formation of a parakeratotic cornified layer in inflamed skin. The epithelial ridges become elongated and broadened, and hyperkeratosis is evident in chronic eczema. The infiltration of dermal lymphocytes and vascular dilatations can be found at all stages. The pathological features in our metal ion-induced ACD mice at seven days after challenge resemble these features. Therefore, our model enables investigation of the precise immune response of metal-allergic inflamed skin.

In terms of the LPS interaction with metal allergy, we identified the accumulation of T cells in the footpads of metal chloride– and LPS-sensitized, metal chloride–challenged mice compared with those of sensitized mice without LPS administration. This finding may suggest that signals from TLR4 induced by LPS stimuli might be required for the induction of metal allergy. Thus, we speculate that LPS is indispensable for the establishment of metal allergy in mice and plays an important role as an adjuvant to induce metal-specific T cells in the inflamed skin of metal ion–induced ACD mice.

Our studies demonstrated successful establishment of a murine model of metal ion-induced ACD with appropriate pathological reactions that reflect the metal ion-specific immune response. In addition, we identified the allergen-specific positive T cells in metal ion-induced ACD mice.

## 3. Immune Responses in Inflamed Metal ACD in Terms of the TCR

We next focused on the accumulation of metal-specific T cells in the inflamed skin. Similar to contact hypersensitivity to classical haptens, the infiltration of lymphocytes into the sites of allergic inflammation is essential for mediating metal allergy. In addition to T cells, mast cells, NK cells, and granulocytes have been shown to be involved in metal-induced inflammation in other animal models [3,4,11,12]. However, the involvement of the antigen specificity and diversity of pathogenic T cells in the development of metal allergy remains unclear.

During an immune response to metal ion–associated antigens, the clonal T cells expansion is observed due to the antigen-specific immune response. T cells bearing TCRs recognize antigens in the form of peptide fragments in relation with major histocompatibility complex (MHC) class I and II molecules on antigen-presenting cells. The high specificity of T cells is determined by the TCRs displayed on their surface, which are heterodimers of an α- and β-chain (Va and Vb) or a γ- and δ-chain (V gamma and V delta). We previously developed an adaptor ligation-mediated polymerase chain reaction (AL-PCR) method that allows TCR repertoires to be defined based on the expression levels of transcripts, even when only small numbers of cells are available [13]. This method enables amplification of all variable regions of the rearranged TCR genes through PCR cycles without skewing. Applying this method to a microplate hybridization assay is simple and reproducible, and enables rapid analysis of TCR repertoires in several diseases in humans and mice [14,15,16]. Random insertions of non-germinal element (N) nucleotides or deletions of nucleotides have been identified in the VN (D) NJ junction region, designated as the complementary determining region 3 (CDR3), and are thought to be responsible for the recognition of antigenic peptide content [17,18]. Thus, any specific recognition of antigens by CDR3 will lead to clonal expansion of T cells. Because CDR3 has different sequences and lengths, it is possible to analyze the diversity of TCRs using a CDR3-size spectratyping method that provides a rapid scan of all TCR Va and Vb-region transcripts grouped according to the utilized V-region gene and chain length [19].

Metal ions can induce the proliferation of human T cells *in vitro*, and limited TCR repertoires have been observed in human T cells from patients with metal allergies [20,21,22]. However, TCR analysis has not been performed in the previous murine models of metal allergy. We hypothesized that restricted TCR usage may reflect the prolonged exposure of the host immune system to putative metal ion–associated antigens. To elucidate the immune responses to metal ion–associated antigens, we examined the characteristics of the T cells within allergic tissue specimens in our models in terms of TCR repertoires and CDR3-size spectratyping. Recently, we successfully identified each metal-specific TCR repertoire in the inflamed skin at the elicitation phase in Pd-, Ni-, and Cr-allergic mice [8,9,10]. Unexpectedly, the TCR repertoire was found to be specific: TCR Va14Ja18 and Vb8.2 were commonly used in BALB/c and C57BL/6 mice by Ni-induced allergic skin. TCR Va14Ja18 and Vb8-2 are a major subset of mouse invariant NK T (iNK T) cells, and we found that iNK T cells accumulated in the inflamed skin of Ni-allergic mice. In Cr allergy, the infiltrating T cells from Cr-induced ACD mice expressed CD4+ and used a specific TCR repertoire expressing TCR Va11-1, Va14-1, Vb8-2, and Vb14-1. In Ni and Cr allergies, we have identified iNK T cells in lymphocytic infiltrates at a high frequency during the elicitation phase. Thus, we suggest that metal-specific T cells driven by invariant NK T cells might contribute to the pathogenesis of metal allergy.

## 4. The Possible Role of NK T Cells in Metal Allergy

NK T cells (also called iNK T cells and Va14 iNK T cells) were originally defined as T cells that expressed CD161c and other receptors typical of NK cells. This definition has subsequently been refined to specify a subpopulation of T cells that express a TCR with an invariant Va14Ja18 TCR Va chain paired with a restricted subset of TCR Vb chains [23]. NK T cells recognize glycolipids (microbial derived or self-glycolipids) presented in the context of CD1d (a MHC class I–like molecule) [24]. After activation, NK T cells produce Th1- and Th2-type molecules such as interferon (IFN)-γ and interleukin-4 to regulate adaptive immune responses [25]. Mature NK T cells in mice are mainly CD4 single-positive or CD4/CD8 double-negative cells. NK T cells predominantly express Vb8.2 joined with a bias of a particular Jb usage including Jb2.1, Jb2.5, or Jb2.7 [26,27].

Recently, NK T cells were also identified in the lesional skin of contact dermatitis (including Ni allergy) in humans [28]. Thus, NK T cells may be involved in the pathogenesis of contact hypersensitivity reactions, possibly by recognizing endogenous antigens such as self-lipids. Water-soluble Ni salts can pass through the ion channels of cell membranes or can be taken up by cells along with other molecules such as proteins and amino acids, so Ni can be incorporated readily into the body [29]. We hypothesized that Ni allergy can be triggered by intracellular Ni that can combine with self-lipids on cellular membranes. We therefore speculate that Ni-modified self-lipids may activate NK T cells in the lesions of Ni-induced dermatitis in our model. A previous study suggested a role of NK T cells in the sensitization phase of contact hypersensitivity (CHS) by promoting the survival and maturation of dendritic cells in draining lymph nodes [30]. The authors indicated that the dendritic cell–NK T cell interaction has a pivotal role in the sensitization phase of CHS. Regarding the cross-reactivity of metal allergy, a previous study suggested that, among the tested ultrapure metals (Ni, Pd, Co, Cr, Cu, and Au), only Ni and Pd cross-reacted [31]. These results may imply that NK T cells participate in the cross-reactivity of metal allergy. A previous study showed that the Th1 subset of NK T cells has a dominant and critical role in allergy. In addition, the immune response in Ni allergy was skewed towards a Th1 response with a minimal Th2 response [32]. The Th1 subset of NK T cells may expand locally in response to Ni.

**Figure 1:**
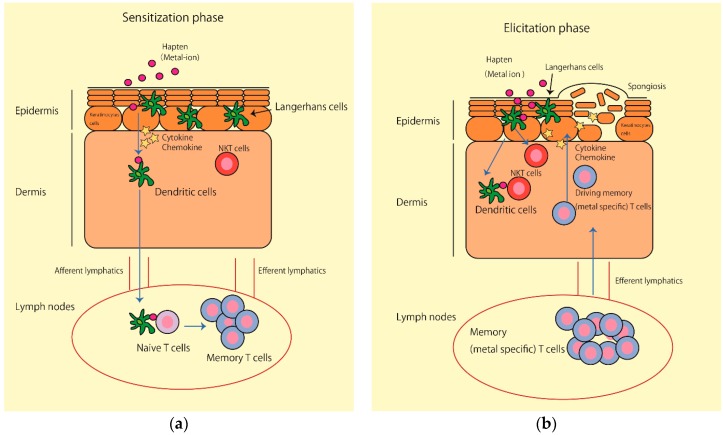
Schematic mechanism of metal ion–induced allergic contact dermatitis. (**a**) Sensitization phase in metal allergy. Step 1: Metal ions form complexes with partner molecules within the body, thereby becoming antigens. A hapten (metal ion) combines with a native protein and activates keratinocytes (KCs), cutaneous Langerhans cells (LCs), and dermal dendritic cells (DCs) through the innate immune system; Step 2: Activated DCs capture antigens, mature, and migrate to the regional lymph nodes via afferent lymphatics; Step 3: Migrated DCs present antigens to naive T cells in draining lymph nodes. NK T cells affect DC functions and regulate the excessive immune response; (**b**) Elicitation phase in metal allergy. Step 1: KCs are activated by re-exposure to haptens and produce various cytokines and chemokines that activate endothelial cells and draining memory metal-specific T cells; Step 2: Infiltrated metal-specific effector T cells are activated and produce proinflammatory cytokines and chemokines that activate KCs and induce further inflammatory cell infiltration; Step 3: NK T cells may regulate the excessive acquired immune response caused by metal-specific effector T cells.

Based on these findings, we summarized the schematic mechanism of metal ion–induced ACD and the possible role of NK T cells in both sensitization and elicitation phases (Figure 1a,b). We propose that NK T cells act as an early amplification step in the innate immune response in the sensitization phase, and bridge to the adaptive immunity in the elicitation phase. We thus speculate that the regulation of excessive NK T cell immune responses might be a new diagnostic or therapeutic target for metal allergy.

## 5. Conclusions and Future Direction

Our novel mouse model is useful for understanding the pathological roles of T cells in metal ion allergy, and the regulation of NK T cells might be involved in the immune responses of metal allergy. Further studies using this mouse model of metal allergy will contribute to the diagnosis of metal allergy in terms of the regulation of NK T cells, as well as to new treatments to control metal-specific T cells.
